# A rapid and efficient method for studies of virus interaction at the host cell surface using enteroviruses and real-time PCR

**DOI:** 10.1186/1743-422X-6-217

**Published:** 2009-12-07

**Authors:** Nina Jonsson, Maria Gullberg, Stina Israelsson, A Michael Lindberg

**Affiliations:** 1 School of Pure and Applied Natural Sciences, University of Kalmar, SE-391 82 Kalmar, Sweden

## Abstract

**Background:**

Measuring virus attachment to host cells is of great importance when trying to identify novel receptors. The presence of a usable receptor is a major determinant of viral host range and cell tropism. Furthermore, identification of appropriate receptors is central for the understanding of viral pathogenesis and gives possibilities to develop antiviral drugs. Attachment is presently measured using radiolabeled and subsequently gradient purified viruses. Traditional methods are expensive and time-consuming and not all viruses are stable during a purification procedure; hence there is room for improvement. Real-time PCR (RT-PCR) has become the standard method to detect and quantify virus infections, including enteroviruses, in clinical samples. For instance, primers directed to the highly conserved 5' untranslated region (5'UTR) of the enterovirus genome enable detection of a wide spectrum of enteroviruses. Here, we evaluate the capacity of the RT-PCR technology to study enterovirus host cell interactions at the cell surface and compare this novel implementation with an established assay using radiolabeled viruses.

**Results:**

Both purified and crude viral extracts of CVB5 generated comparable results in attachment studies when analyzed with RT-PCR. In addition, receptor binding studies regarding viruses with coxsackie- and adenovirus receptor (CAR) and/or decay accelerating factor (DAF) affinity, further demonstrated the possibility to use RT-PCR to measure virus attachment to host cells. Furthermore, the RT-PCR technology and crude viral extracts was used to study attachment with low multiplicity of infection (0.05 × 10^-4^TCID_50_/cell) and low cell numbers (250), which implies the range of potential implementations of the presented technique.

**Conclusion:**

We have implemented the well-established RT-PCR technique to measure viral attachment to host cells with high accuracy and reproducibility, at low cost and with less effort than traditional methods. Furthermore, replacing traditional methods with RT-PCR offers the opportunity to use crude virus containing extracts to investigate attachment, which could be considered as a step towards viral attachment studies in a more natural state.

## Background

The first critical step in the viral lifecycle involves attachment and entry via interactions with one or several cell surface receptors. The presence of a suitable receptor is the main determinant of viral host range, cell tropism and pathogenesis [1,2]. Enteroviruses form one genus within the family *Picornaviridae *[3] and are important human pathogens causing a wide spectrum of clinical symptoms including meningitis, myocarditis, gastroenteritis, poliomyelitis, common cold and diabetes [4]. The enterovirus genome is a positive single stranded RNA molecule of approximately 7.500 nucleotides starting with a 5'untranslated region (5'UTR) followed by an open reading frame encoding a polyprotein of about 2.200 amino acids and a 3'UTR ending with a poly A tail [5]. Several cellular receptors have been identified as attachment molecules for *Picornaviridae*, including the poliovirus receptor (PVR) [6], various types of integrins [7-10], intracellular adhesion molecule 1 (ICAM-1) [11,12], decay-accelerating factor (DAF or CD55) [13,14] and coxsackie- and adenovirus receptor (CAR) [15,16]. Group B coxsackieviruses (CVB) with its six serotypes, CVB1-6, may enter the susceptible cell by attachment to CAR, a 46-kDa transmembrane protein that also serves as a receptor for many adenoviruses [17]. In addition, some strains of CVB1, 3 and 5 can interact with an additional receptor, DAF, a 70-kDa regulatory protein consisting of four short consensus repeats (SCRs) [18]. CVBs can attach to DAF, but are usually unable to enter the cell in the absence of CAR [19,20] unless the DAF receptors are cross-linked by specific anti-DAF monoclonal antibodies (MAbs) [21]. Thus, binding to DAF is a characteristic feature of many enteroviruses including enterovirus 70 and echovirus 7 [13,14,21-25]. Interactions between a virus and the host cell surface are generally studied using purified radiolabeled virions that are allowed to attach to cultured cells.

The real-time PCR (RT-PCR) technology utilizes the standard PCR method with the addition of measuring the accumulation of amplified DNA in real-time by a fluorescent signal. RT-PCR uses the threshold cycle (Ct) value, *i.e*. the lowest number of cycles necessary to detect a fluorescent signal above a threshold, to quantify amplified DNA. The recorded Ct value is directly proportional to the starting number of cDNA, *i. e*. viral RNA, where one cycle theoretically represents the double amount of template. RT-PCR is the method of choice to detect and quantify virus infections in clinical samples, including enteroviruses [26,27]. Amplification of highly conserved regions of the enterovirus 5'untranslated region (5'UTR) is the golden standard to detect enteroviruses in specimens [28,29].

In this report, we demonstrate for the first time the possibility to use RT-PCR to study interactions between enteroviruses and their target cells. RT-PCR is a rapid and sensitive method suitable for attachment studies and allows the use of crude virus containing extracts as well as limited amounts of cells and viruses.

## Methods

### Cells and viruses

HeLa-SoH (provided by M. Rovainen, Helsinki, Finland), CHO, CHO-CAR and CHO-DAF [30,31] cells were maintained in DMEM (Sigma), supplemented with 10% newborn calf serum (NCS) (Biological Industries) and 1% penicillin-streptomycin and L-glutamine (Sigma). 1mg/ml G418 (Sigma) was added to CHO-CAR cells and 0.75mg/ml Hygromycin B (Invitrogen) to CHO-DAF cells. The clinical isolate CVB5 strain 151rom70 was kindly provided by T. Hovi (Helsinki, Finland), while echovirus 7 strain *Wallace *(EV7W, ATCC VR-37) and CVB2 strain *Ohio *(CVB2O, ATCC VR-29) were obtained from American Tissue Culture Collection (ATCC). Viruses were propagated and titrated on GMK cells.

### Binding assays

CVB5 151rom70 was labeled by growth in GMK cells in the presence of ^35^S-methionine and ^35^S-cysteine (Perkin-Elmer). Virions were purified by sucrose gradient centrifugation as described elsewhere [8]. Binding assays, using both purified radiolabeled viruses and crude virus extracts, were carried out in suspension as described by Arnberg *et al*. [32]. Briefly, cells were detached with versene solution, pelleted and washed twice in binding buffer (DMEM supplemented with 2% NCS and 1% penicillin-streptomycin and L-glutamine). Cells and viruses, 2.5 × 10^5 ^cells per tube if not stated otherwise, were incubated for 2 h on ice or at room temperature and washed twice with ice-cold or room temperatured binding buffer before re-suspension in 200 μl serum-free media, all in triplicates. For measures of radioactivity, the radiation was determined by liquid scintillation counting, while non-radioactive samples were frozen for further applications.

### Two-step RT-PCR

RNA was extracted using QIAamp viral RNA extraction kit (Qiagen) according to the manufacturer's instructions and used for reverse transcription. cDNA synthesis was performed using Applied Biosystems TaqMan reverse transcriptase kit according to the manufacturer's protocol. Assay conditions for quantification of extracted viral RNA were optimized using the Applied Biosystems 7500 Real-Time PCR System (Applied Biosystems), by using a two-step RT-PCR and SYBR Green detection method as previously described [33]. Obtained Ct values were recalculated into RNA copies, *i.e*. virions, by the use of a standard curve previously described by Jonsson *et al*. [33].

### Statistical analyses

Individual data pairs were analysed by the unpaired t test, and one-way analysis of variance followed by Dunnetts post-test was used to compare groups vs. controls. Data were considered statistically significant if p < 0.05.

## Results and Discussion

### Comparison of purified and unpurified viruses

Interactions between a virus and the target cell are generally studied using radioactive labeling, and subsequently gradient purification of viruses. The purified and labeled viruses are allowed to interact with cultured cells and the amount of bound radioactivity is used as a measurement of the viral attachment capacity. In this article, we demonstrate that RT-PCR is an alternative, rapid and efficient method to study viral interaction with the cell surface. RT-PCR can complement or replace the expensive and time-consuming methods presently employed.

**Figure 1:**
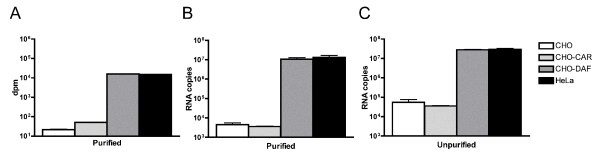
**Attachment studies using a clinical isolate of CVB5**. A) 3 × 10^4^dpm (corresponding to MOI 0.05 TCID_50_/cell) was incubated with 2.5 × 10^5 ^cells in suspension at 4°C for 2 h. Attachment was measured using scintillation counting. B) MOI 0.05 TCID_50_/cell of the labeled, purified virus and C) MOI 0.05 TCID_50_/cell of unpurified virus incubated as described in A) and the amount of virus bound to the cell surface was measured with a two-step RT-PCR. Results are presented as mean ± SEM.

A clinical isolate of CVB5, CVB5 151rom70, was analysed for validation and comparison between the standard method with labeled viruses and the RT-PCR, with the assumption that one enterovirus genome is equivalent to one virion. 3 × 10^4 ^dpm ^35^S-labeled virus (corresponding to a multiplicity of infection (MOI) of 0.05 TCID_50_/cell) or MOI 0.05 TCID50/cell of crude virus extracts were incubated with cells in suspension on ice for 2 h. The attached virus was measured using scintillation counting (purified, radiolabeled virus) or RT-PCR (purified and crude virus extracts). Using radiolabeled viruses (Figure 1A) the clinical isolate of CVB5 demonstrated similar affinity for HeLa and CHO-DAF, which could be expected due to approximately equal amount of expressed DAF on the cell surfaces determined by flow cytometry (data not shown). Comparable results were recorded using RT-PCR (Figure 1B), thus supporting the applicability of RT-PCR in viral affinity measurements. In addition, the interaction between CVB5 151rom70 and the cell surface were studied using crude virus extracts (Figure 1C). Although the binding of CVB5 to CHO cells was higher using crude extracts, the specific binding to HeLa and recombinant CHO-DAF cells were statistical significant compared to CHO cells.

The relative binding (fold difference) of viruses attached to HeLa in comparison to CHO was calculated for the results presented in Figure 1. Considering the purified virus, the RT-PCR present ~3000 fold difference (Figure 1B), while the difference in dpm is ~700 fold (Figure 1A). Crude virus give a ~550 fold difference (Figure 1C), which demonstrates that unpurified viruses and RT-PCR give results that are comparable with labeled and purified viruses, but with less effort and expense.

Altogether, these results clearly indicate the capacity of the RT-PCR technology in studies of viral attachment. Furthermore, RT-PCR gives the opportunity to study viruses that are not purified through differential ultracentrifugation and therefore enables attachment studies using virus in a more natural environment. Binding assays described and discussed from this point were therefore carried out using crude viral extracts.

The data obtained did not distinguish between binding to CHO and CHO-CAR using the clinical isolate of CVB5, although an indication of attachment to CHO-CAR was observed when measuring interactions at the cell surface using purified viruses (Figure 1A). CVB prototype strains have been shown to utilize CAR as receptor [15,34]. Several low-passage clinical isolates of CVB5 are less affected by antibodies directed against CAR [23], indicating that some CVB strains may use alternate receptors. Attachment of crude virus extracts, analysed by RT-PCR, showed a higher proportion of binding to CHO than labeled viruses, which is consistent with previous reports. Martino *et al*. [35] showed that several unpurified isolates of CVB have an affinity for CHO cells. Newcombe *et al*. [36] reported that unpurified coxsackievirus A20 (CVA20) binds to RD cells, cells that do not express its major receptor ICAM-1, whereas labeled and subsequently purified CVA20 have no affinity to RD cells. Similar observations were reported by Pash *et al*. [31], where two strains of CVB3 demonstrated no affinity for CHO cells when purified virions were used, while both strains interacted with CHO and HeLa cells at comparable levels when crude virus extracts were used. Hence, our observations that a higher degree of binding to CHO cells is obtained with crude viruses are in accordance with these previous reports. These observations by others and us indicate that gradient purification may alter the structure of the virion or remove components that affect the interactions with the cell surface. The observed differences suggest that the interaction between virus and host cell differs in an environment that resembles infection in its natural state in comparison to the highly purified radiolabeled virus. Hence the use of RT-PCR that enables to measure attachment without any purification could be an advantage.

### Studies regarding binding to DAF and CAR

**Figure 2:**
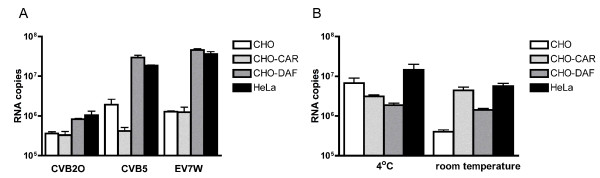
**Binding studies for DAF and CAR**. A) MOI 0.5 TCID_50_/cell ofCVB2O, EV7W and CVB5 was incubated with 2.5 × 10^5 ^cells in suspension for 2 h at 4°C and virus attachment was subsequently measured using RT-PCR. B) MOI 5 TCID_50_/cell of CVB2O was incubated as described in A) at 4°C or at room temperature and attached virus was measured by RT-PCR. Results are presented as mean ± SEM.

Due to the fact that binding to DAF, but not to CAR, was recorded using the experimental conditions described above and RT-PCR, two additional well-characterized enterovirus serotypes were included in the study, EV7W and CVB2O. EV7W utilizes DAF as receptor [13,14] and CVB2O uses CAR [15,16,34,35]. In a first set of experiments, MOI 0.5 TCID_50_/cell of each virus were allowed to attach to HeLa, CHO, CHO-CAR and CHO-DAF cells at 4°C. EV7W and CVB5 demonstrated equal binding to HeLa and CHO-DAF, a result in accordance with previous reports [20,37]. However, the RT-PCR analyses of CVB2O attachment showed no statistical significant difference in attachment between any of the cell lines at 4°C (Figure 2A). The observed lower affinity of CVB2O to susceptible cells could be due to the fact that DAF is ten times more abundant than CAR on HeLa cells [38], and that CVB2O previously has been shown to have a significantly slower attachment rate to cells than CVB5 [39]. The increase of MOI (MOI 5 TCID_50_/cell) of CVB2O and attachment carried out at 4°C or at room temperature (Figure 2B) showed that increasing the temperature is an important parameter using our experimental setup. Binding of CVB2O to CHO cells was reduced at room temperature demonstrating that incubation at a higher temperature reduced unspecific attachment of this virus to CHO cells, while attachment to CHO-CAR, CHO-DAF and HeLa remained at the same level. Thus, a significant difference in attachment to CHO-CAR and HeLa in comparison to CHO was observed performing the measurements at room temperature.

Interestingly, these studies indicate that CVB2O has some affinity to CHO-DAF cells, thus suggesting that CVB2O may have a capacity to interact with DAF at the cell surface. Although, presented results give indications of an affinity for DAF that was not affected by temperature, this novel finding needs to be further explored. Further investigation using the RT-PCR method to quantify binding of CVB2O and CVB4 may reveal that these viruses have some affinity for DAF, which may explain why CVB2O could be adapted to cytolytic replication in RD cells despite the absence of CAR [40].

### Using RT-PCR for sensitive studies of viral host cell interactions

**Figure 3:**
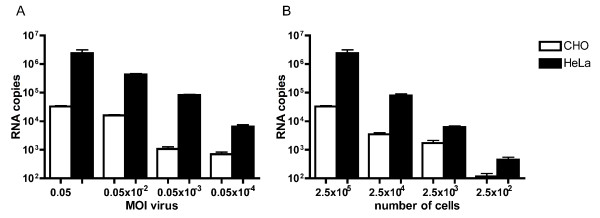
**Sensitivity studies to measure viral attachment with RT-PCR**. A) Decreasing amount of virus, MOI 0.05-0.05 × 10^-4 ^TCID_50_/cell, was incubatedwith 2.5 × 10^5 ^cells and bound virus was analysed with RT-PCR. B) Decreasing number of CHO and HeLa cells, 2.5 × 10^5^-2.5 × 10^2^, were incubated with MOI 0.05 TCID_50_/cell of CVB5 and attached virus was measured with RT-PCR. Results are presented as mean ± SEM.

To explore the implementations of RT-PCR in viral binding studies the limitations in MOI and cell number that could be used to generate recordable affinities using SYBR green and two-step RT-PCR were investigated. A fixed number of HeLa and CHO cells (2.5 × 10^5^) were incubated with various amounts of CVB5, starting with MOI 0.05 TCID_50_/cell to a final ratio of MOI 0.05 × 10^-4 ^TCID_50_/cell.A significant difference in binding to HeLa in comparison to CHO could be observed even at a MOI of 0.05 × 10^-4 ^TCID_50_/cell (Figure 3A). These results demonstrate the potential to measure specific interactions with very low amounts of viruses using this RT-PCR approach.

By using a fixed ratio of virus/host cell relation (MOI 0.05 TCID_50_/cell), the ability to study interaction using a limited amount of cells was also investigated. A decreasing number of cells were incubated with CVB5 at a concentration of MOI 0.05 TCID_50_/cell and significant differences in binding to HeLa in comparison with CHO cells were observed with as few as 250 cells (Figure 3B). The data presented above clearly demonstrate that RT-PCR is a valuable technology for studies of interactions between virus and cells even when low amounts of cells or viruses are available.

Investigating viral binding using labeled and purified viruses is usually conducted with high virus to cell ratio, high concentration of cells and virus in a small sample volume [31,32,37,41]. We have demonstrated, using well-characterized enteroviruses, that RT-PCR is a powerful method to quantify interactions between a virus and the cell surface. The options to use unpurified viruses, low MOI and cell numbers indicate the opportunity to study virus host cell interaction even when the amount of cells and viruses are limited.

## Conclusion

This article describes a straightforward, rapid and robust method that with high accuracy can be used to quantify viral attachment, as an alternative to traditional methods. We present data that demonstrate the opportunity to use crude virus extracts and RT-PCR to study binding of viruses to cells. This is an important step towards studying viruses in their natural state rather than using highly purified viruses for these types of studies. The potential to circumvent purification and radiolabeling of viruses gives the possibility to study viral attachment with less effort and at low cost. In addition, it gives the opportunity to investigate binding of viruses that are not stable during the purification process by differential ultracentrifugation and viruses that can not be cultured in cell culture.

## Competing interests

The authors declare that they have no competing interests.

## Authors' contributions

NJ planned the experimental setup, prepared virus stocks, carried out the binding assays, RNA extraction and real-time PCR analysis. NJ drafted and wrote the manuscript. MG developed the protocol for the binding assay, radiolabeled and purified the clinical isolate and performed the radioactive measured binding assay. SI participated in handling and analysing recombinant cell lines. AML was involved in the study design, draft and revision of manuscript. All authors have read and approved the final manuscript.

## References

[B1] Rossmann MG He Y Kuhn RJ Picornavirus-receptor interactions Trends Microbiol 2002 10 324 331 10.1016/S0966-842X(02)02383-1 12110211

[B2] Evans DJ Almond JW Cell receptors for picornaviruses as determinants of cell tropism and pathogenesis Trends Microbiol 1998 6 198 202 10.1016/S0966-842X(98)01263-3 9614344

[B3] Fauquet CM Mayo MA Maniloff J Desselberger U Bell LA Virus Taxonomy 2005 Elsevier Academic Press

[B4] Pallanch MA Roos RP Enteroviruses: polioviruses, coxsackieviruses, ehcoviruses and newer enteroviruses 2001 fourth Philadelphia, PA: Lippincott, Williams and Wilkins

[B5] Racianello VR Picornaviridae: The viruses and their replication 2001 4 Philadelphia: Lippincott Williams & Wilkins

[B6] Mendelsohn CL Wimmer E Racaniello VR Cellular receptor for poliovirus: molecular cloning, nucleotide sequence, and expression of a new member of the immunoglobulin superfamily Cell 1989 56 855 865 10.1016/0092-8674(89)90690-9 2538245

[B7] Bergelson JM Shepley MP Chan BM Hemler ME Finberg RW Identification of the integrin VLA-2 as a receptor for echovirus 1 Science 1992 255 1718 1720 10.1126/science.1553561 1553561

[B8] Bergelson JM St John N Kawaguchi S Chan M Stubdal H Modlin J Finberg RW Infection by echoviruses 1 and 8 depends on the alpha 2 subunit of human VLA-2 J Virol 1993 67 6847 6852 8411387 10.1128/jvi.67.11.6847-6852.1993 PMC238130

[B9] Roivainen M Piirainen L Hovi T Virtanen I Riikonen T Heino J Hyypia T Entry of coxsackievirus A9 into host cells: specific interactions with alpha v beta 3 integrin, the vitronectin receptor Virology 1994 203 357 365 10.1006/viro.1994.1494 7519807

[B10] Nelsen-Salz B Eggers HJ Zimmermann H Integrin alpha(v)beta3 (vitronectin receptor) is a candidate receptor for the virulent echovirus 9 strain Barty J Gen Virol 1999 80 Pt 9 2311 2113 10501481 10.1099/0022-1317-80-9-2311

[B11] Greve JM Davis G Meyer AM Forte CP Yost SC Marlor CW Kamarck ME McClelland A The major human rhinovirus receptor is ICAM-1 Cell 1989 56 839 847 10.1016/0092-8674(89)90688-0 2538243

[B12] Staunton DE Dustin ML Erickson HP Springer TA The arrangement of the immunoglobulin-like domains of ICAM-1 and the binding sites for LFA-1 and rhinovirus Cell 1990 61 243 254 10.1016/0092-8674(90)90805-O 1970514

[B13] Bergelson JM Chan M Solomon KR St John NF Lin H Finberg RW Decay-accelerating factor (CD55), a glycosylphosphatidylinositol-anchored complement regulatory protein, is a receptor for several echoviruses Proc Natl Acad Sci USA 1994 91 6245 6248 10.1073/pnas.91.13.6245 7517044 PMC44175

[B14] Ward T Pipkin PA Clarkson NA Stone DM Minor PD Almond JW Decay-accelerating factor CD55 is identified as the receptor for echovirus 7 using CELICS, a rapid immuno-focal cloning method Embo J 1994 13 5070 5074 7525274 10.1002/j.1460-2075.1994.tb06836.x PMC395453

[B15] Bergelson JM Cunningham JA Droguett G Kurt-Jones EA Krithivas A Hong JS Horwitz MS Crowell RL Finberg RW Isolation of a common receptor for Coxsackie B viruses and adenoviruses 2 and 5 Science 1997 275 1320 1323 10.1126/science.275.5304.1320 9036860

[B16] Tomko RP Xu R Philipson L HCAR and MCAR: the human and mouse cellular receptors for subgroup C adenoviruses and group B coxsackieviruses Proc Natl Acad Sci USA 1997 94 3352 3356 10.1073/pnas.94.7.3352 9096397 PMC20373

[B17] Roelvink PW Lizonova A Lee JG Li Y Bergelson JM Finberg RW Brough DE Kovesdi I Wickham TJ The coxsackievirus-adenovirus receptor protein can function as a cellular attachment protein for adenovirus serotypes from subgroups A, C, D, E, and F J Virol 1998 72 7909 7915 9733828 10.1128/jvi.72.10.7909-7915.1998 PMC110119

[B18] Nicholson-Weller A Decay accelerating factor (CD55) Curr Top Microbiol Immunol 1992 178 7 30 1385047 10.1007/978-3-642-77014-2_2

[B19] Bergelson JM Mohanty JG Crowell RL St John NF Lublin DM Finberg RW Coxsackievirus B3 adapted to growth in RD cells binds to decay-accelerating factor (CD55) J Virol 1995 69 1903 1916 7531780 10.1128/jvi.69.3.1903-1906.1995 PMC188804

[B20] Shafren DR Bates RC Agrez MV Herd RL Burns GF Barry RD Coxsackieviruses B1, B3, and B5 use decay accelerating factor as a receptor for cell attachment J Virol 1995 69 3873 3877 7538177 10.1128/jvi.69.6.3873-3877.1995 PMC189108

[B21] Shafren DR Viral cell entry induced by cross-linked decay-accelerating factor J Virol 1998 72 9407 9412 9765499 10.1128/jvi.72.11.9407-9412.1998 PMC110371

[B22] Powell RM Schmitt V Ward T Goodfellow I Evans DJ Almond JW Characterization of echoviruses that bind decay accelerating factor (CD55): evidence that some haemagglutinating strains use more than one cellular receptor J Gen Virol 1998 79 Pt 7 1707 1713 9680134 10.1099/0022-1317-79-7-1707

[B23] Bergelson JM Modlin JF Wieland-Alter W Cunningham JA Crowell RL Finberg RW Clinical coxsackievirus B isolates differ from laboratory strains in their interaction with two cell surface receptors J Infect Dis 1997 175 697 700 9041347 10.1093/infdis/175.3.697

[B24] Karnauchow TM Dawe S Lublin DM Dimock K Short consensus repeat domain 1 of decay-accelerating factor is required for enterovirus 70 binding J Virol 1998 72 9380 9383 9765493 10.1128/jvi.72.11.9380-9383.1998 PMC110365

[B25] Philipson L Bengtsson S Brishammar S Svennerholm L Zetterqvist O Purification And Chemical Analysis Of The Erythrocyte Receptor For Hemagglutinating Enteroviruses Virology 1964 22 580 590 10.1016/0042-6822(64)90080-7 14166119

[B26] Verstrepen WA Bruynseels P Mertens AH Evaluation of a rapid real-time RT-PCR assay for detection of enterovirus RNA in cerebrospinal fluid specimens J Clin Virol 2002 25 Suppl 1 S39 43 10.1016/S1386-6532(02)00032-X 12091080

[B27] Verstrepen WA Kuhn S Kockx MM Vyvere ME Van De Mertens AH Rapid detection of enterovirus RNA in cerebrospinal fluid specimens with a novel single-tube real-time reverse transcription-PCR assay J Clin Microbiol 2001 39 4093 4096 10.1128/JCM.39.11.4093-4096.2001 11682535 PMC88492

[B28] Mohamed N Elfaitouri A Fohlman J Friman G Blomberg J A sensitive and quantitative single-tube real-time reverse transcriptase-PCR for detection of enteroviral RNA J Clin Virol 2004 30 150 156 10.1016/j.jcv.2003.08.016 15125871

[B29] Dierssen U Rehren F Henke-Gendo C Harste G Heim A Rapid routine detection of enterovirus RNA in cerebrospinal fluid by a one-step real-time RT-PCR assay J Clin Virol 2008 42 58 64 10.1016/j.jcv.2007.11.016 18164234

[B30] Selinka HC Wolde A Pasch A Klingel K Schnorr JJ Kupper JH Lindberg AM Kandolf R Comparative analysis of two coxsackievirus B3 strains: putative influence of virus-receptor interactions on pathogenesis J Med Virol 2002 67 224 233 10.1002/jmv.2211 11992583

[B31] Pasch A Kupper JH Wolde A Kandolf R Selinka HC Comparative analysis of virus-host cell interactions of haemagglutinating and non-haemagglutinating strains of coxsackievirus B3 J Gen Virol 1999 80 Pt 12 3153 3158 10567646 10.1099/0022-1317-80-12-3153

[B32] Arnberg N Edlund K Kidd AH Wadell G Adenovirus type 37 uses sialic acid as a cellular receptor J Virol 2000 74 42 48 10.1128/JVI.74.1.42-48.2000 10590089 PMC111511

[B33] Jonsson N Gullberg M Lindberg AM Real-time polymerase chain reaction as a rapid and efficient alternative to estimation of picornavirus titers by tissue culture infectious dose 50% or plaque forming units Microbiol Immunol 2009 53 149 154 10.1111/j.1348-0421.2009.00107.x 19302525

[B34] Hsu KH Lonberg-Holm K Alstein B Crowell RL A monoclonal antibody specific for the cellular receptor for the group B coxsackieviruses J Virol 1988 62 1647 1652 2451756 10.1128/jvi.62.5.1647-1652.1988 PMC253193

[B35] Martino TA Petric M Weingartl H Bergelson JM Opavsky MA Richardson CD Modlin JF Finberg RW Kain KC Willis N The coxsackie-adenovirus receptor (CAR) is used by reference strains and clinical isolates representing all six serotypes of coxsackievirus group B and by swine vesicular disease virus Virology 2000 271 99 108 10.1006/viro.2000.0324 10814575

[B36] Newcombe NG Andersson P Johansson ES Au GG Lindberg AM Barry RD Shafren DR Cellular receptor interactions of C-cluster human group A coxsackieviruses J Gen Virol 2003 84 3041 3050 10.1099/vir.0.19329-0 14573809

[B37] Spiller OB Goodfellow IG Evans DJ Almond JW Morgan BP Echoviruses and coxsackie B viruses that use human decay-accelerating factor (DAF) as a receptor do not bind the rodent analogues of DAF J Infect Dis 2000 181 340 343 10.1086/315210 10608785

[B38] Goodfellow IG Evans DJ Blom AM Kerrigan D Miners JS Morgan BP Spiller OB Inhibition of coxsackie B virus infection by soluble forms of its receptors: binding affinities, altered particle formation, and competition with cellular receptors J Virol 2005 79 12016 12024 10.1128/JVI.79.18.12016-12024.2005 16140777 PMC1212587

[B39] Crowell RL Comparative generic charactericstics of picornavirus-receptor interactions 1976 New York: Raven Press

[B40] Polacek C Ekstrom JO Lundgren A Lindberg AM Cytolytic replication of coxsackievirus B2 in CAR-deficient rhabdomyosarcoma cells Virus Res 2005 113 107 115 10.1016/j.virusres.2005.04.021 15964091

[B41] Milstone AM Petrella J Sanchez MD Mahmud M Whitbeck JC Bergelson JM Interaction with coxsackievirus and adenovirus receptor, but not with decay-accelerating factor (DAF), induces A-particle formation in a DAF-binding coxsackievirus B3 isolate J Virol 2005 79 655 660 10.1128/JVI.79.1.655-660.2005 15596863 PMC538729

